# Optimizing the accuracy of cortical volumetric analysis in traumatic brain injury

**DOI:** 10.1016/j.mex.2020.100994

**Published:** 2020-07-11

**Authors:** Bram R. Diamond, Christine L. Mac Donald, Aina Frau-Pascual, Samuel B. Snider, Bruce Fischl, Kristen Dams-O'Connor, Brian L. Edlow

**Affiliations:** a Center for Neurotechnology and Neurorecovery, Department of Neurology, Massachusetts General Hospital and Harvard Medical School, Boston, MA; b Athinoula A. Martinos Center for Biomedical Imaging, Department of Radiology, Massachusetts General Hospital and Harvard Medical School, Charlestown, MA; c Department of Neurological Surgery, University of Washington, Seattle, WA; d Harvard-MIT Health Sciences and Technology, Computer Science and Artificial Intelligence Laboratory, Massachusetts Institute of Technology, Cambridge, MA; e Department of Rehabilitation and Human Performance, Icahn School of Medicine at Mount Sinai, New York, NY; f Department of Neurology, Icahn School of Medicine at Mount Sinai, New York, NY

**Keywords:** Traumatic brain injury, Contusion, Mri, FreeSurfer, Neurodegeneration

## Abstract

Cortical volumetric analysis is widely used to study the anatomic basis of neurological deficits in patients with traumatic brain injury (TBI). However, patients with TBI-related lesions are often excluded from MRI analyses because cortical lesions may compromise the accuracy of reconstructed surfaces upon which volumetric measurements are based. We developed a FreeSurfer-based lesion correction method and tested its impact on cortical volume measures in 87 patients with chronic moderate-to-severe TBI. We reconstructed cortical surfaces from T1-weighted MRI scans, then manually labeled and removed vertices on the cortical surfaces where lesions caused inaccuracies. Next, we measured the surface area of lesion overlap with seven canonical brain networks and the percent volume of each network affected by lesions.

• The lesion correction method revealed that cortical lesions in patients with TBI are preferentially located in the limbic and default mode networks (95.7% each), with the limbic network also having the largest average surface area (4.4+/−3.7%) and percent volume affected by lesions (12.7+/−9.7%).
• The method has the potential to improve the accuracy of cortical volumetric measurements and permit inclusion of patients with lesioned brains in MRI analyses.
• The method also provides new opportunities to elucidate network-based mechanisms of neurological deficits in patients with TBI.

Specifications Table

**Table:** 

Subject Area	Neuroscience
More specific subject area	Traumatic Brain Injury, Neuroimaging
Method name	FreeSurfer Lesion Correction
Name and reference of original method	FreeSurfer cortical volume measurements Original method detailed in the 2000 issue of the Proceedings of the National Academy of Sciences of the United States of America [1].
Resource availability	https://github.com/freesurfer/freesurfer https://surfer.nmr.mgh.harvard.edu/fswiki https://github.com/ComaRecoveryLab/Lesion_Correction

## Method details

### Background

Cortical volumetric analysis with FreeSurfer [1,2] is widely used to study the neuroanatomic basis of cognitive, behavioral, and motor deficits in patients with traumatic brain injury (TBI) [3], [4], [5], [6]. However, cortical lesions caused by TBI pose major challenges to FreeSurfer's standard automated magnetic resonance imaging (MRI) processing pipeline. Lesions often compromise the accuracy of the cortical surfaces that are reconstructed and used by FreeSurfer to generate volumetric measurements [4,^6^,7]. As a result, TBI imaging studies have historically excluded patients with large focal lesions [5,8]. Development of a tool that accounts for lesions in cortical volumetric analysis is needed to prevent the systematic exclusion of patients with large cortical lesions and to ensure that TBI imaging studies are generalizable across the full spectrum of cortical pathology. Moreover, integration of such a tool into the FreeSurfer software platform would create new opportunities to study network-based mechanisms of disease [9,10] using canonical atlases [11].

Here, we propose a novel FreeSurfer-based lesion correction method and illustrate its impact on cortical volumetric measures in patients with chronic TBI. The lesion correction method differs in several ways from the standard FreeSurfer approach to editing reconstructed cortical surfaces. Standard cortical segmentation using FreeSurfer relies on the assumption that the brain has normal anatomy and that any surface inaccuracies are related to the FreeSurfer processing pipeline. However, in patients with cortical lesions caused by TBI, FreeSurfer's reconstruction of the cortical surface can be grossly inaccurate due to focal encephalomalacia and distorted anatomy. This methodological limitation of the standard FreeSurfer editing approach is the main motivation for the lesion correction method proposed here. The new method makes no assumptions about lesioned cortical surface anatomy, and it minimizes bias by requiring the manual rater simply to identify inaccuracies without changing the surfaces. In this study, we use the lesion correction method to assess the topology of lesion overlap with functional brain networks and to characterize inter-network differences in lesion burden. We also distribute the lesion correction method to the academic community to facilitate future studies of network-based mechanisms of neurological deficits in patients with TBI.

### Patients

**Fig. 1:**
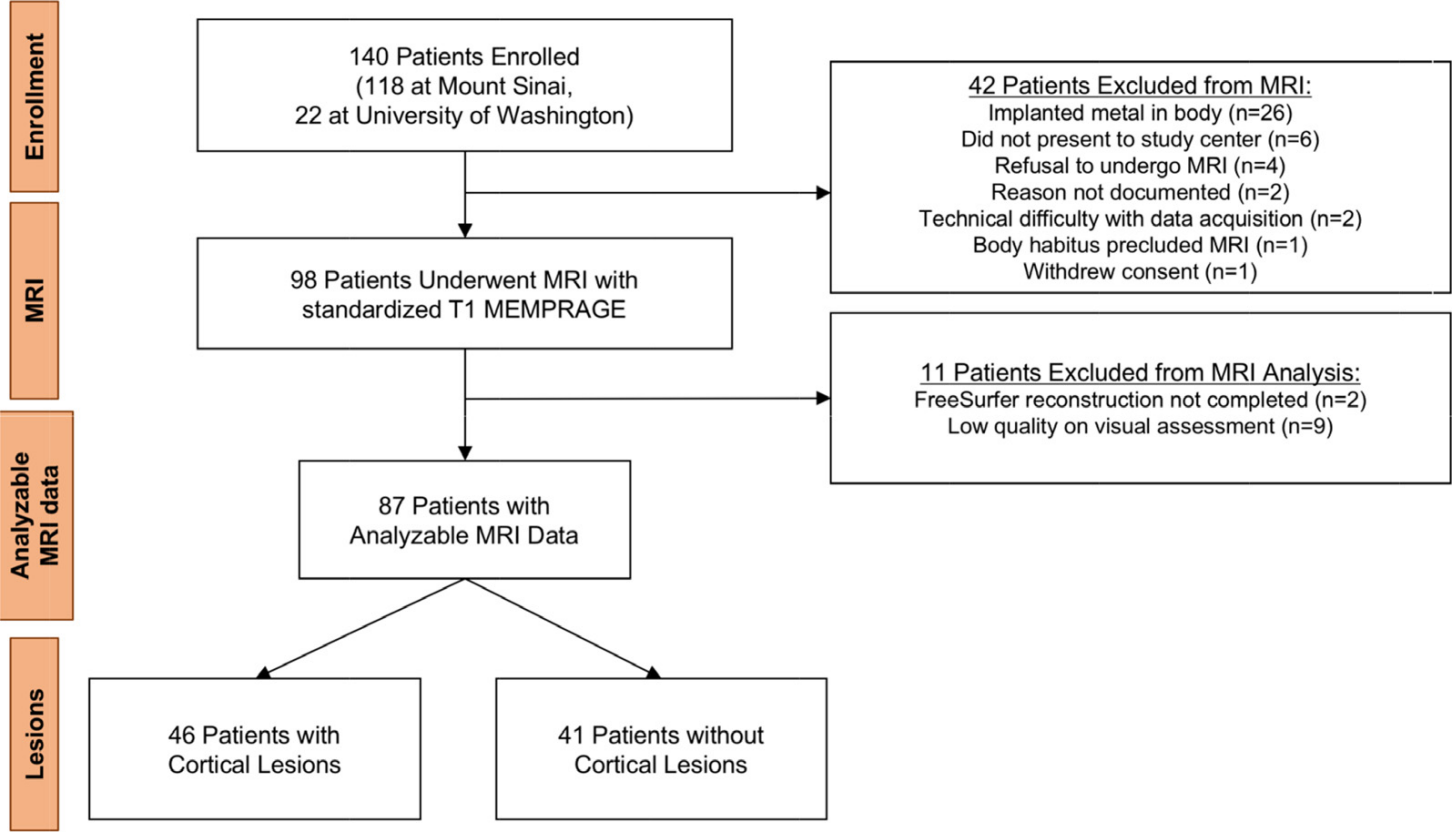
CONSORT Diagram.

Between May 2014 and January 2019, we prospectively enrolled 141 patients with a history of TBI at two academic medical centers as part of the Late Effects of TBI (LETBI) study [12]. Patients were included if they had sustained a moderate-to-severe TBI at least one year prior to enrollment. We characterized TBI according to the Department of Defense classification system [13] based on all available information, including medical records, radiology reports, and self-report as elicited by structured lifetime TBI screening questionnaire (the Brain Injury Screening Questionnaire; BISQ) [14]. When duration of unconsciousness was not known and records were not available to confirm the presence of intracranial abnormality, severity was coded as missing. Of the 141 enrolled participants, 98 completed an MRI scan (see CONSORT diagram in Fig. 1).

### MRI data acquisition

Patients at Mount Sinai were scanned using a Siemens Skyra (Siemens Medical Solutions, Erlangen, Germany) 3 Tesla (T) MRI scanner with a 32-channel head coil for signal reception, and patients at University of Washington were scanned using a Philips Achieva 3T MRI scanner with a 32-channel head coil. Patients underwent standardized MRI using a T1-weighted multi-echo MPRAGE (MEMPRAGE) [15] sequence with 1 mm isotropic voxels. All LETBI sequences were designed to maximize consistency with the National Institutes of Health Common Data Elements for TBI Neuroimaging [16].

### MRI processing

We first processed all MEMPRAGE data using the standard FreeSurfer pipeline (version 6.0) for cortical surface reconstruction and cortical volume estimation [2]. We used the “big ventricles” option to optimize automatic segmentation for a patient population with enlarged ventricles. In accordance with FreeSurfer recommended best practices (https://surfer.nmr.mgh.harvard.edu/fswiki/FsTutorial/TroubleshootingDataV6.0), we visually inspected output files, made manual edits to the white matter segmentation, and added control points. To ensure that the lesion correction method would be tested in an unbiased manner, we did not manually edit regions bordering cortical lesions. We then resampled the Yeo 7-Network resting-state functional connectivity atlas [11] onto each patient's reconstructed cortical surface using FreeSurfer's surface-based registration tool (https://surfer.nmr.mgh.harvard.edu/fswiki/mri_surf2surf) [17].

Given that T2-weighted Fluid Attenuated Inversion Recovery (FLAIR) data may improve the cortical surface reconstruction and thereby minimize the need for manual edits, users who have access to 1 mm isotropic resolution T2 FLAIR data can incorporate these data into FreeSurfer's reconstruction pipeline using the multimodal option (-FLAIRpial).

### Quality assessments

We performed visual quality assessment for all 98 scans based upon delineation of gray-white matter boundaries and the accuracy of the FreeSurfer-generated surfaces. We defined scan quality using an integer scale: 0 = scan excluded because FreeSurfer failed to complete the processing pipeline; 1 = scan excluded because surface inaccuracies would have required major manual edits; 2 = scan included because only minor manual edits required; 3 = scan included without requiring manual edits. For any scan that received a score of 1 by the primary rater (B.R.D.), a second rater (B.L.E.) reviewed the scan to achieve consensus. Our primary method for determining scan inclusion was qualitative visual assessment because inaccurate FreeSurfer-based segmentations can confound quantitative measurements. Nevertheless, we performed quantitative assessments of signal-to-noise ratio (SNR) and contrast-to-noise ratio (CNR) and tested for correlations with visual assessments of scan quality.

Due to the presence of severe anatomic distortions, two of the 98 patients’ scans did not complete FreeSurfer's standard processing pipeline (visual assessment scores=0). To explore the relationship between visual and quantitative assessments of scan quality, we measured the SNR and CNR in the remaining 96 scans that completed the FreeSurfer reconstruction process. We calculated SNR using the white-matter (WM) segmentation, and we calculated CNR as the average of the WM-GM and GM-cerebrospinal fluid contrasts (see https://github.com/ComaRecoveryLab/Lesion_Correction for specific commands). We tested the hypothesis that the quantitative SNR and CNR scores differed between the groups of scans that received visual assessment scores of 1, 2, and 3.

Of the 96 scans, nine received a visual assessment score of 1 by the two raters and were excluded, yielding a final sample size of 87 patients. All 87 patients were assigned a visual quality score of 2, indicating the need for minor FreeSurfer editing (e.g. editing of the brain mask and use of control points). A two-tailed T-test demonstrated that the quantitative SNR but not the CNR values differed between the 87 patients included in the analysis and the 9 patients excluded (*p*<0.0001 and *p* = 0.65, respectively). A summary of the SNR and CNR values for the scans that were excluded versus the scans that were included is provided in Table 1, considering both sites together and separately. We also found SNR and CNR values of included patients not differing across sites (*p* = 0.54 and *p* = 0.09, respectively). Note that all the excluded subjects were from the site Mount Sinai School of Medicine (MSSM), and that when considering only this site, the same conclusions hold.

**Table 1:** Quantitative Assessment of MRI Data at Each Enrollment Site.

Enrollment Site	Cohort	SNR	CNR
MSSM + UW	Inclusion Cohort (*n* = 87)	15.2 +/− 3.3	1.0 +/− 0.3
	Exclusion Cohort (*n* = 9)*	8.3 +/− 2.6	0.9 +/− 0.2
	P value	<0.0001	0.65
MSSM	Inclusion Cohort (*n* = 66)	15.1 +/− 2.9	1.0 +/− 0.4
	Exclusion Cohort (*n* = 9)*	8.3 +/− 2.6	0.9 +/− 0.2
	P value	<0.0001	0.9
UW	Inclusion Cohort (*n* = 21)	15.6 +/− 4.5	1.1 +/− 0.1
	Exclusion Cohort (*n* = 0)	–	–
	P value	–	–

⁎ For two subjects, the FreeSurfer processing pipeline could not be completed. Thus, only 9 of the 11 excluded patients could undergo signal-to-noise ratio (SNR) and contrast-to-noise ratio (CNR) analysis. P values were determined using two-tailed T-tests. MSSM = Mount Sinai School of Medicine; UW = University of Washington.

### Patient demographics and clinical characteristics

The 87-patient cohort was comprised of 60.9% men, with a mean +/− SD age of 56.7 +/− 12.0 years. Injury severity was classified as mild (*n* = 3), moderate (*n* = 42), and severe (*n* = 32); in 10 participants, the duration of loss of consciousness (LOC) was unknown and records were not available. The duration from most recent TBI to MRI was 10.9 +/− 9.1 years. Additional clinical and demographic data are provided in Table 2, and Table 3.

**Table 2:** Patient Clinical and Demographic Characteristics.

	Patients with Cortical Lesions (*n* = 46)	Patients without Cortical Lesions (*n* = 41)	Patients with Unanalyzable MRI (*n* = 11)	Patients Excluded from MRI (*n* = 42)
Age (mean +/− SD years)	58.5 +/− 11.2	54.8 +/− 12.8	61.3 +/− 10.4	57.1 +/− 14.8
Sex (M/F)	26/20	27/14	8/3	26/16
Years from Most Recent TBI to MRI (mean +/− SD)	10.1 +/− 11.2	11.9 +/− 10	16.5 +/− 16.1	Not applicable

**Table 3:** Patient Demographics at the Two Enrollment Sites.

	Mount Sinai	University of Washington
Age (mean +/− SD years)	54.1 +/− 12.3	65.0 +/− 5.6
Gender (F/M)	28/38	6/15
Race*	0/5/57/2/2	2/0/19/0/0
Ethnicity⁎⁎	52/11/2/1	18/2/0/1

⁎ American Indian or Alaska Native/Black or African-American/White/Unknown/Not Reported.

⁎⁎ Not Hispanic or Latino/Hispanic or Latino/Not Reported/Unknown.

**Fig. 2:**
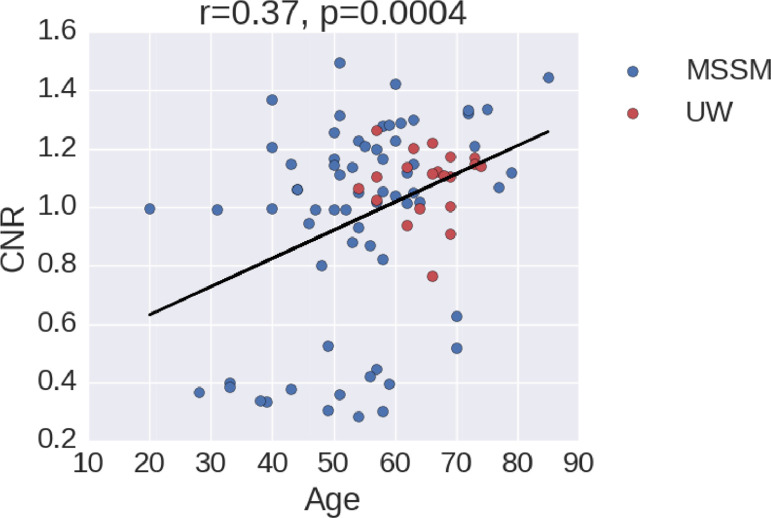
Age versus CNR for the subjects at each institution: Mount Sinai School of Medicine (MSSM; blue dots) and University of Washington (UW; red dots). The black line shows the linear regression of all the points, with *r* = 0.37 and p-value < 0.001. Here CNR was computed as the average of white/gray matter and gray matter/cerebrospinal fluid contrasts, averaged across hemispheres. More details on CNR calculation are available at https://github.com/ComaRecoveryLab/Lesion_Correction.

Of note, Fig. 2 shows that age has an impact on CNR and therefore white/gray matter and gray matter/cerebrospinal fluid differentiation. This age-effect on CNR may impact the segmentation results when running FreeSurfer. The correlation between CNR and age is positive, an unexpected finding given that a negative correlation has previously been observed [18]. However, the cloud of points is not well defined and has different clusters. Furthermore, there was no difference in CNR between scans that were included in the analysis and excluded (see Table 1), suggesting that the association between age and CNR did not affect patient inclusion in this study.

### Lesion identification and classification

**Fig. 3:**
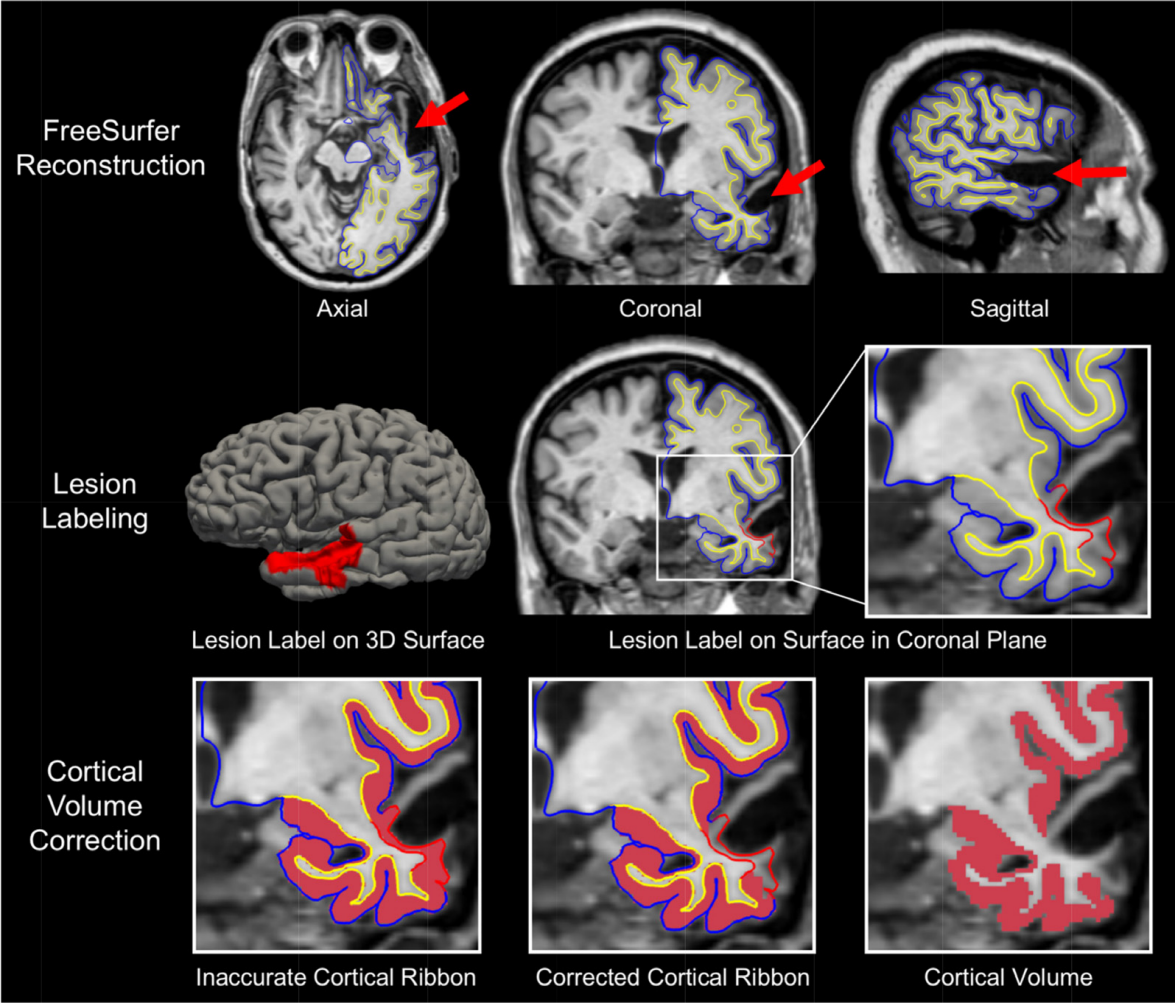
Overview of Lesion Correction Method.

We next assessed each MEMPRAGE scan for focal lesions causing encephalomalacia of the cerebral cortex (Fig. 3, top row) [16]. All such lesions were considered for subsequent lesion correction analysis and classified according to the cortical network(s) with which they overlapped. To ensure robust and reproducible methods for lesion identification, we performed an inter-rater reliability analysis among three investigators who identified lesions in a randomly selected group of 20 MRI scans and calculated lesion volumes using the standard ABC/2 method [19]. Two investigators were board-certified neurologists with fellowship training in Neurocritical Care (B.L.E. and S.B.S.) and one was a research technician (B.R.D.).

Row 1: Axial, coronal, and sagittal T1-weighted images of a representative patient with traumatic brain injury. FreeSurfer reconstructions of the cortical surface (blue line) and gray-white surface (yellow line) are used to visually identify regions where a cortical lesion (red arrows) caused surface inaccuracies. Row 2: We manually outlined lesions by labeling inaccurate vertices on the cortical surface (left image). This surface inaccuracy (labeled in red) is shown in the coronal plane in the middle image and the right, zoomed image. The red label passes through lesioned, encephalomalacic tissue. Row 3: To correct for the inaccuracy in the surface label at the site of the lesion, we remove the volume of cortex within the lesion label and perform cortical volumetric measures that exclude the lesioned tissue.

### Interrater reliability

We used the intraclass correlation coefficient, as implemented in R (The R Foundation, https://www.r-project.org), to test interrater reliability for lesion volume measurements. The intraclass coefficient between the two physician raters across 20 datasets was 0.99 [95% Confidence Interval 0.98, 0.99]. The intraclass coefficients between the physician raters and the technician rater for these same datasets were 0.95 [0.91, 0.97] and 0.96 [0.93, 0.98], respectively. Because sufficient inter-rater reliability was established in this test set (*n* = 20; intraclass correlation coefficient > 0.9), all subsequent lesion identification was performed by the technician rater, B.R.D.

### Lesion correction – methodological principles

Generating cortical volume measurements requires FreeSurfer to model two surfaces, the gray matter (GM) surface and the white matter (WM) surface. Each surface mesh is comprised of thousands of vertices, with 1:1 pairings of vertices between the two surfaces. The distance between these two surfaces at any given vertex-pair provides a measure of cortical thickness. To compute the volume of a cortical region (such as a network of the 7-Network Yeo atlas), FreeSurfer computes the average regional thickness and then multiplies that value by the region's surface area. Thus, any defect or inaccuracy in these surfaces will yield inaccurate volumetric measures.

Historically, the standard method for improving the FreeSurfer-generated GM and WM surfaces requires a trained research technician to edit certain output volumes (e.g. brainmask.mgz & wm.mgz) from the recon-all automated pipeline and then regenerate surfaces using the updated volumes. Because these are the volumes from which the surfaces are modeled, manually correcting them improves the anatomic accuracy of the GM and WM surface meshes. However, there are fundamental limitations to how this approach can improve the GM and WM surfaces. Given that FreeSurfer is designed to analyze brains with normal anatomy, these editing approaches are helpful for minimizing surface inaccuracies caused by suboptimal scans (e.g. motion blurring, poor CNR, non-isotropic voxels, MR-induced artefacts, etc.). In patients with cortical lesions, such as the patients studied here, the lesions create surface inaccuracies that are attributable to anatomic distortions and encephalomalacia, independent of inaccuracies related to image quality. When these cortical lesions are present, the GM and WM boundaries can be undetectable on T1-weighted images (as shown in Fig. 3), even when using a high-resolution T1 sequence. Hence, any FreeSurfer-generated measurement of the cortical thickness is baseless. Manually editing the correct output files from recon-all can prompt surface models to move closer to a rater's desired location, but it is unlikely that the resulting cortical volume measures would be anatomically accurate or biologically valid. Furthermore, the FreeSurfer-based cortical volumetric measurements depend on a continuous surface mesh, thereby assuming a complete and undamaged cortex. In patients with lesions caused by TBI, there may be encephalomalacic regions of cortex that disrupt cortical continuity. In short, when cortical lesions blur the GM and WM boundary or cause significant cortical atrophy, the standard methods for improving surfaces cannot be used to improve the accuracy of cortical volume measurements.

This methodological limitation is the main motivation for the lesion correction method proposed here. The lesion correction method is not meant to replace the standard approach to manually improving FreeSurfer outputs detailed above. Rather, it is intended to be used after manual editing, as an additional tool for MRI scans with lesion-induced surface inaccuracies. The proposed method makes no assumptions about the GM and WM surface anatomy at sites of cortical lesions, and it minimizes bias by requiring the manual rater simply to identify inaccurate surfaces without changing the surfaces in a subjective manner. The method maintains the continuity of the reconstructed FreeSurfer surface mesh while also accounting for regions of cortical atrophy.

### Lesion correction protocol and guide

The newly proposed lesion correction method involves the following steps, which can be performed by a research technician in less than 60 min of active time per patient. The example we have provided can be applied to any surface parcellation already registered to a subjects’ FreeSurfer surfaces. Notably, this method does not require the addition of a T2-weighted scan. Nevertheless, incorporating a T2-weighted image may reduce the time needed for manual edits [20]. If the research team has access to a T2-weighted image for every patient, then we suggest amending step (1) by adding either the “-T2pial” or the “-FLAIRpial” option and making use of the T2 contrast in step (5). If the research team has already run recon-all and performed the classic manual edits, skip to step (3). All code relating to the steps described below is distributed at https://github.com/ComaRecoveryLab/Lesion_Correction.

(1) Process the patient's MRI through FreeSurfer's standard recon-all pipeline. If applicable, use the “-bigventricles” option to improve anatomical segmentation for patients with pathologically enlarged ventricles.
(2) Spend approximately 30 min assessing FreeSurfer output and applying manual edits where necessary before re-running recon-all to apply the adjustments. The addition of a T2-weighted image may reduce the time needed for manual edits. Repeat this step as needed. For additional details on troubleshooting a FreeSurfer output, see the FreeSurfer Guide to Recon Editing https://surfer.nmr.mgh.harvard.edu/fswiki/FsTutorial/TroubleshootingData.
(3) Using the Development Version of FreeView (free download available https://surfer.nmr.mgh.harvard.edu/pub/dist/freesurfer/dev), load the patient's T1.mgz and pial surface files. Turn the curvature off and hide 3D slices. A template terminal command is provided here:

freeview \

-v $SUBJECTS_DIR/<Subject ID>/mri/T1.mgz \

-f $SUBJECTS_DIR/<Subject ID>/surf/lh.pial:curvature_method=off \

-f $SUBJECTS_DIR/<Subject ID>/surf/rh.pial:curvature_method=off \

-hide-3d-slices

(4) Select the “1 & 3 Horizontal” layout from the view panel selection on the top toolbar. This can also be called in the command displayed above by including “-layout 4″ at the end.
(5) Scroll through the T1.mgz (and T2raw.mgz or FLAIRraw.mgz, if applicable) to identify any reconstructed surfaces that pass through cortical lesions (see the Fig. 3 for example). Make note of these lesions, as you will manually label each one in the following steps.
(6) Left-click on the “Custom Fill” button located under the loaded surface files on the left-hand toolbar. Then, select “Make Path” and left-click the 3D surface render to place points outlining the lesion on the ??.pial surface (where “??” stands for “rh” or “lh” depending on the hemisphere). It is very important that these points should maintain an unbroken chain and only be placed on a single hemisphere.
(7) Once the point-outline is complete, left-click on the “Make Closed Path” button to connect the points.
(8) Now that a lesion path has been created, remove the points by left-clicking “Clear Marks”. Left-click to create a single point anywhere inside the closed path and then left-click “Custom Fill” (followed by “Fill”, when prompted) to fill the lesion label (Fig. 3, middle row).
(9) The label will now be filled and a new label file (“label_?”) will appear in the Label Index on the left-hand toolbar. Left-click “Save” and assign the label a new name with the prefix “lh.” or “rh.” to specify the hemisphere, followed by “lesion-??.label” (where “??” denotes a 0-padded integer between 00 and 99) to note the number of previous labels on that hemisphere. For example, the first lesion labeled on the left hemisphere of any given subject will be named, “lh.lesion-01.label”. Repeat this step for every cortical lesion that has interfered with either of the pial surfaces.
(10) The “correct_segstats.sh” wrapper script provided on GitHub should automatically combine your labels for each subject's hemisphere into overlays (“??.all-lesions.mgz”) used to correct the volumetric statistics calculated by FreeSurfer. The script combines each subject's label files into a single annotation file for each hemisphere and then converts the annotation files into overlay files using the “mris_label2annot” and “mris_annotation2label” commands, respectively. Then, the script applies the “mris_anatomical_stats” command to extract the cortical volume measurements and ignore any anatomical region within the lesion overlay file. The script will then curate each subjects stats file into two stats tables (one for each hemisphere) by implementing the “aparcstats2table” command.

### Implementation of the lesion correction procedure

To implement the procedure, we visually identified sites where FreeSurfer's modeled surface mesh erroneously passed through subcortical tissue (Fig. 3, top row). Next, we manually labeled these surface-points to produce lesion-induced inaccuracy labels (Fig. 3, middle row). Finally, we applied these labels as exclusion masks to remove affected surface regions and calculate corrected cortical volumes (Fig. 3, bottom row). For a detailed description, please see Lesion Correction Protocol and Guide, above.

**Fig. 4:**
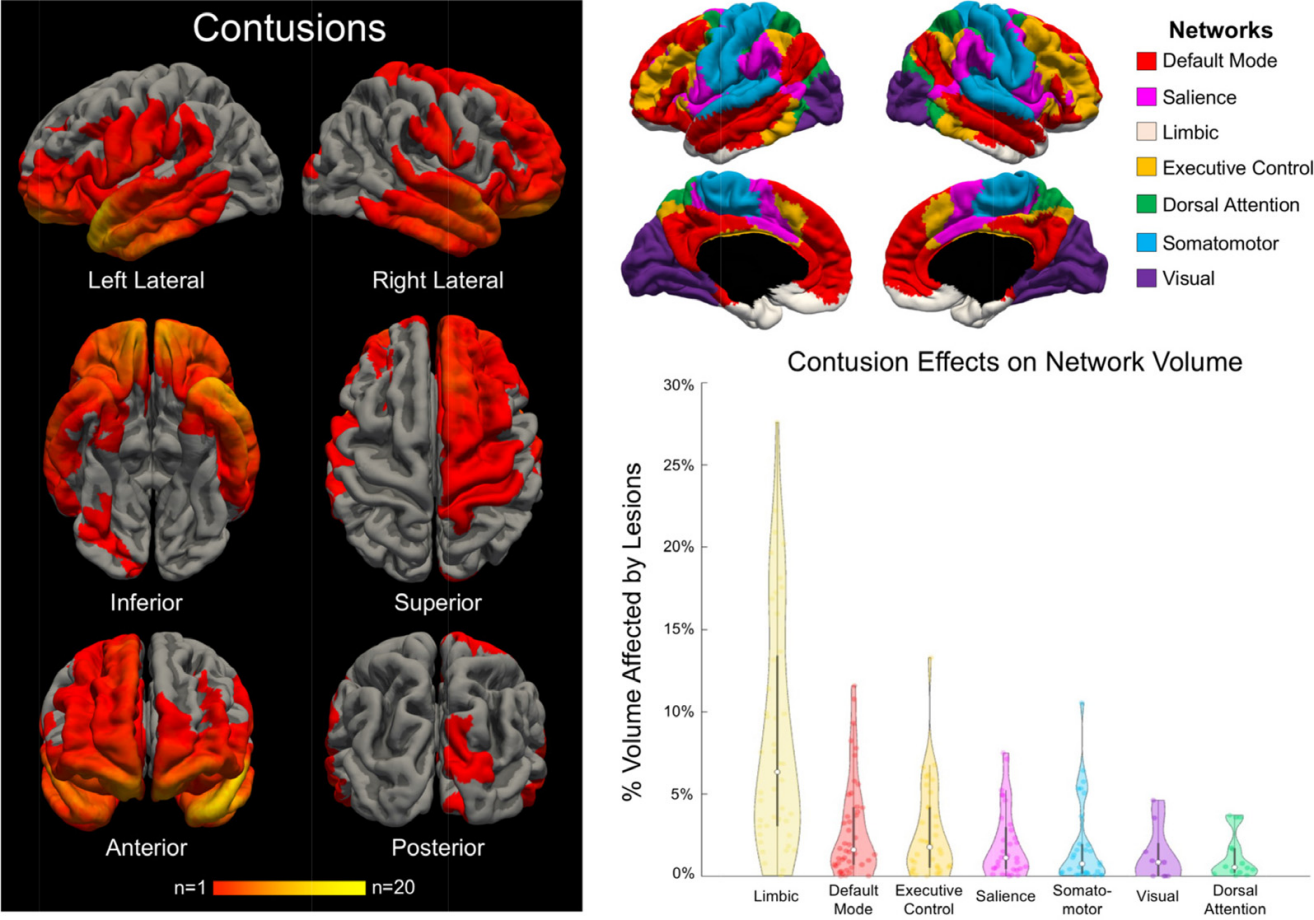
Lesion Topology and Network-based Lesion Effects on Cortical Volume.

After performing this lesion correction procedure, we used standard FreeSurfer tools to measure the average surface area overlap of lesion-induced inaccuracies with each network of the Yeo 7-Network atlas [11] and the average percent volume change of each network caused by the lesion correction procedure (Fig. 4). There was no need to correct cortical volume measurements by total intracranial volume in this study because all network-based measures (i.e.% change in volume) were calculated at the single-subject level.

In the left panel, we show a heat map of cortical lesions for all 46 patients who had at least one lesion. The anatomic regions most commonly affected by cortical lesions were the frontal and temporal lobes, particularly the frontal poles, temporal poles and orbitofrontal regions. In the top right panel, we show the 7 functional networks from the Yeo atlas [11] that were used to investigate network-specific lesion effects. In the bottom right panel, we show a violin plot demonstrating the changes in average cortical volume for each network after applying the lesion correction method. Lesion effects on average cortical volume varied between networks, with the limbic network showing the largest magnitude of decline in average cortical volume after application of the lesion correction method.

### Lesion characteristics and anatomic distribution

Forty-six of the 87 patients had at least one lesion that affected the accuracy of the FreeSurfer-modeled cortical surface. There were 120 total lesions, with a median of 2 lesions per patient (range 1 to 10). On average, lesions overlapped with 4.6 +/− 1.6 of the 7 networks. A group-level lesion topology map demonstrated an orbitofrontal and anterior temporal predominance of the lesions (Fig. 4, Videos 2 and 3).

### Network-based cortical surface area measures

**Fig. 5:**
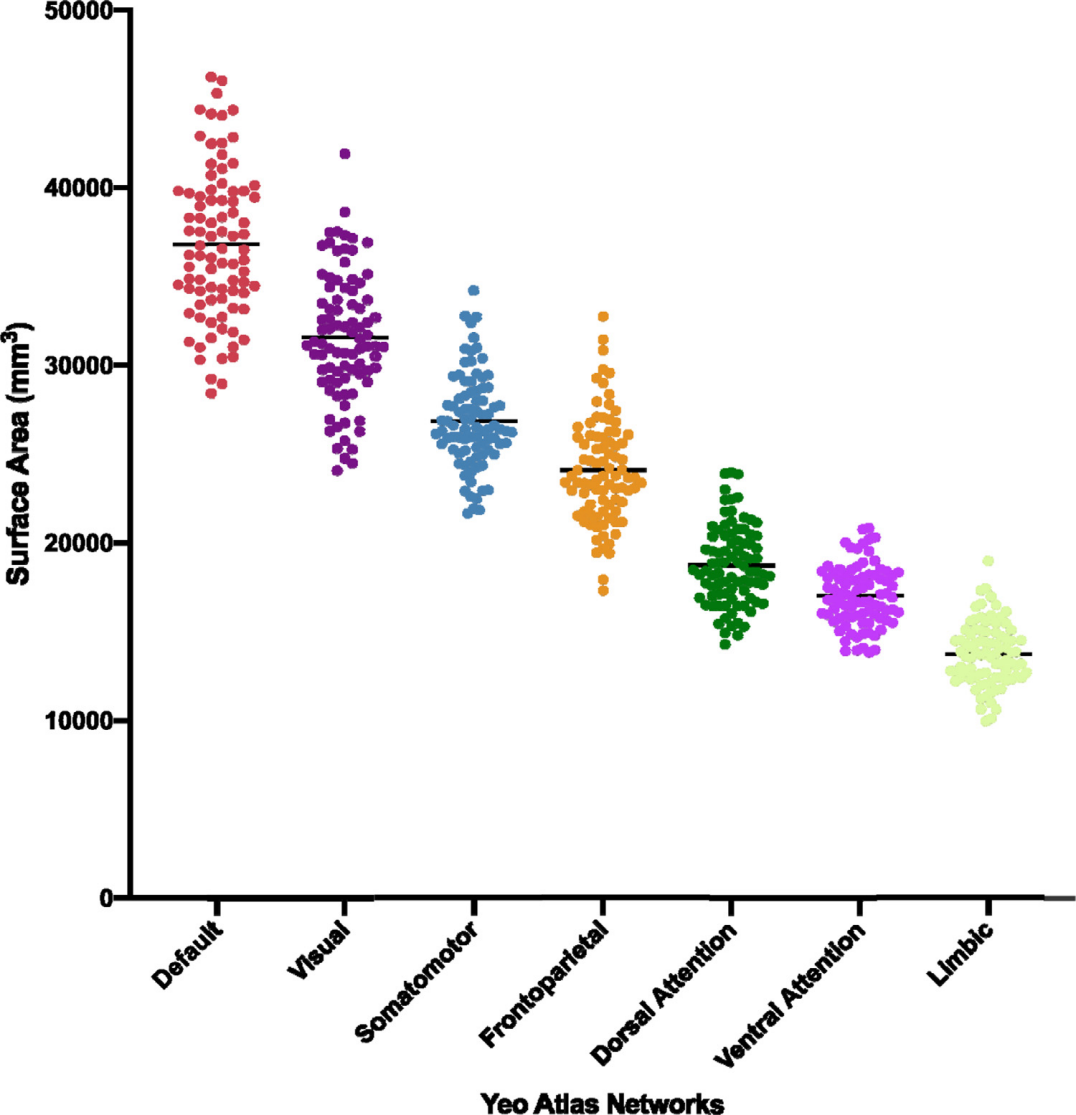
Average Surface Area Measures for the 7 Yeo Functional Networks Across all Patients.

The limbic and default mode networks were the most commonly lesioned, with each network lesioned in 44/46 scans (95.7% incidence). The executive control network was lesioned in 78.3% and the salience network in 71.7% of the 46 scans with lesions. The large limbic lesion burden was observed despite the limbic network having the smallest average surface area of the seven functional networks across all patients (Fig. 5). The largest mean percentage of lesion-network surface area overlap occurred within the limbic network (4.4 +/− 3.7% of total network surface area; Table 4).

**Table 4:** Network Overlap with Cortical Lesions.

Network	Number of Lesions Within Network (*n* = 374)*	Number of Patients with Lesioned Network (of 46 Patients with Cortical Lesions)	Average% of Network Surface Area Affected by Overlapping Lesions
Default Mode	93	44 (95.7%)	1.4 +/− 2.1
Salience	60	33 (71.7%)	1.2 +/− 2.0
Limbic	89	44 (95.7%)	4.4 +/− 3.7
Dorsal Attention	20	16 (34.8%)	0.8 +/− 1.2
Executive Control	58	36 (78.3%)	1.6 +/− 2.4
Somatomotor	45	31 (67.4%)	1.3 +/− 2.1
Visual	9	9 (19.6%)	1.3 +/− 1.7

% lesion overlap data are reported as mean +/− SD.

⁎ The total number of lesions within all networks is greater than 120 because most lesions overlapped with more than one network. On average, lesions overlap with mean +/− SD 4.6 +/− 1.6 of the 7 networks.

The limbic network has the smallest average surface area of the 7 networks, yet it is the network that is most commonly affected by cortical lesions in patients with chronic moderate-to-severe traumatic brain injury.

### Network-based cortical volume measures

When considering networks impacted by the lesion correction method in the 46 patients with cortical lesions, we observed a median decrease in network-based cortical volume of 3.4% (range <1.0% to 47.0%). The limbic network had the largest lesion-induced mean +/− SD percentage decrease in cortical volume (12.7 +/− 9.7%; Table 5).

**Table 5:** Network-specific Effects of Cortical Lesions on Average Cortical Volumetric Measures.

Network	Pre-Correction Cortical Volume (ml)	Post-Correction Cortical Volume (ml)	% Change in Cortical Volume*
Default Mode (*n* = 44)	67.4 +/− 25.8	70.6 +/− 26.2	5.3 +/− 6.1
Salience (*n* = 33)	30.8 +/− 12.6	31.5 +/− 12.4	3.8 +/− 4.7
Limbic (*n* = 44)	26.4 +/− 11.5	30.1 +/− 13.0	12.7 +/− 9.7
Dorsal Attention (*n* = 16)	31.7 +/− 12.1	31.9 +/− 12.0	2.2 +/− 3.0
Executive Control (*n* = 36)	39.5 +/− 15.8	40.8 +/− 16.0	4.5 +/− 5.5
Somatomotor (*n* = 31)	44.6 +/− 17.3	45.6 +/− 17.1	3.7 +/− 5.4
Visual (*n* = 9)	49.5 +/− 18.6	49.7 +/− 18.7	1.8 +/− 2.1

This table summarizes the percent volume change in each network caused by the lesion correction method. The average volume (ml) for each network of the 7-Network Yeo Atlas is provided for the 46 patients with cortical lesions. The limbic network was disproportionately lesioned compared to other networks due to the frontotemporal distribution of cortical lesions, as visualized in Fig. 4.

⁎ Measurements for the percent change in cortical volume include networks that have been impacted by the volume correction method. See column 2 of Table 4 for the number of paired (pre vs. post) volumes that were used to calculate each network's percent change in cortical volume.

### Summary and applications

We introduce a new FreeSurfer-based method for cortical volumetric analysis in patients with lesions caused by TBI. We apply this method in a cohort of 87 patients with chronic moderate-to-severe TBI and show that lesion-induced cortical inaccuracies are not equally distributed within the brain's functional networks. Rather, inaccuracies preferentially affected the limbic network, an observation consistent with prior pathology [21,22] and MRI [23] studies showing that traumatic contusions commonly affect the orbitofrontal and temporal nodes of the limbic network. Implementation of the proposed lesion correction method will prevent the systematic exclusion of patients with cortical lesions from MRI volumetric studies and improve the generalizability of MRI studies across the full spectrum of cortical pathology.

These findings demonstrate the potential utility of the new lesion correction method for studying network-based mechanisms of cognitive, behavioral, and motor deficits in patients with TBI. For example, lesion-induced cortical volume changes within the limbic, default mode, and frontoparietal networks (the three most frequently lesioned networks) can be tested for correlations with symptoms that are putatively attributable to their dysfunction, such as behavioral dysregulation, altered self-awareness, and executive dysfunction, respectively. The motivation for this network-based analytic approach is a recent paradigm shift in our field, whereby cognitive and functional deficits are mapped to network disconnections, rather than to focal lesions [9,10]. The method may thus be used to study lesion pathophysiology in patients with chronic TBI by reporting not just the anatomic localization of each lesion, but also its network overlap. The tool can also be used to investigate the functional specificity of structural lesions, including those that injure “hub” nodes at sites of network integration [24]. From a phenomenological standpoint, the application of the new lesion correction tool to large clinical-radiological-pathological databases being acquired by the LETBI [12], Transforming Research and Clinical Knowledge in TBI (TRACK-TBI) [25], Collaborative European NeuroTrauma Effectiveness Research in Traumatic Brain Injury (CENTER-TBI) [26], and other studies, has potential to elucidate pathological signatures of TBI phenotypic classification, with implications for clinical trial selection [27] and prognostication [10].

Several limitations should be considered when interpreting the results of this study. The lesion correction method relies upon an assumption whose validity is difficult to test: we assume that at sites of tissue distortion and encephalomalacia, the cortex is non-functional and therefore should be masked, or removed, from subsequent cortical volume measurements. This assumption is made with the recognition that definitive determination of the functional status of lesioned cortex is not possible solely with T1-weighted MEMPRAGE data. Nevertheless, the assumption that lesioned cortex is non-functional in the population studied here is strongly supported by visual inspection of the data, which reveals complete or near complete absence of cerebral cortex, as shown in Fig. 3. In future multimodal experiments, the lesion correction method can be refined by analyzing the functional properties of lesioned cortex (e.g. with functional MRI or EEG). In future work, it may also be possible to integrate the semi-automated lesion correction method with complementary lesion detection tools, such as convolutional neural networks [28], multi-atlas label propagation [29], multimodal segmentation in 3D Slicer [30] or clustering of image intensities [31].

In considering how the semi-automated lesion correction tool reported here could complement, and add to, the results of previously described automated lesion identification tools, it is important to distinguish between lesion identification and cortical surface correction. Our tool requires visual identification of lesions (hence, the “semi”-automation), which is a potential limitation compared to automated approaches. However, automated approaches may be more likely to miss lesions [32]. Indeed, in the absence of a gold-standard for premortem lesion detection [33], automated tools often rely upon visual inspection when testing their validity [29]. Furthermore, an advantage of our tool is that it goes beyond lesion detection by correcting the effects of lesions on the cortical surface, an application that is directly relevant to future studies investigating correlations between cortical volumetrics and cognitive deficits [34]. Unlike prior tools, our tool can be used to measure point-wise and region-wise estimates of cortical thickness in unlesioned cortex by masking inaccurate regions of cortex.

Another methodological consideration is that the lesion detection method presented here was designed to account for chronic lesions that disrupt the cortical surface, as opposed to acute cortical lesions or small subcortical lesions that do not disrupt the cortical surface. Unlike small subcortical lesions, which are best detected by susceptibility-weighted, T2-weighted, or diffusion-weighted imaging [35,36], the large cortical lesions studied here are readily detectable by T1-weighted imaging, particularly because encephalomalacia in the chronic setting causes profound tissue distortions. In contrast, the potential applicability of this tool to patients with acute cortical lesions should not be assumed. Acute traumatic lesions often have mixed signal characteristics on T1-, T2-, and susceptibility-weighted imaging due to concurrent hemorrhage and edema; as such, acute lesions may be more challenging to visually identify than the chronic lesions studied here. Furthermore, the anatomic boundaries of acute lesions with respect to the cortical surface are more challenging to delineate than are the boundaries of chronic lesions [34]. The application of this tool for acute lesion correction thus requires further testing in the acute TBI population. We anticipate that distribution of the code will enable such testing by the academic community.

**Fig. 6:**
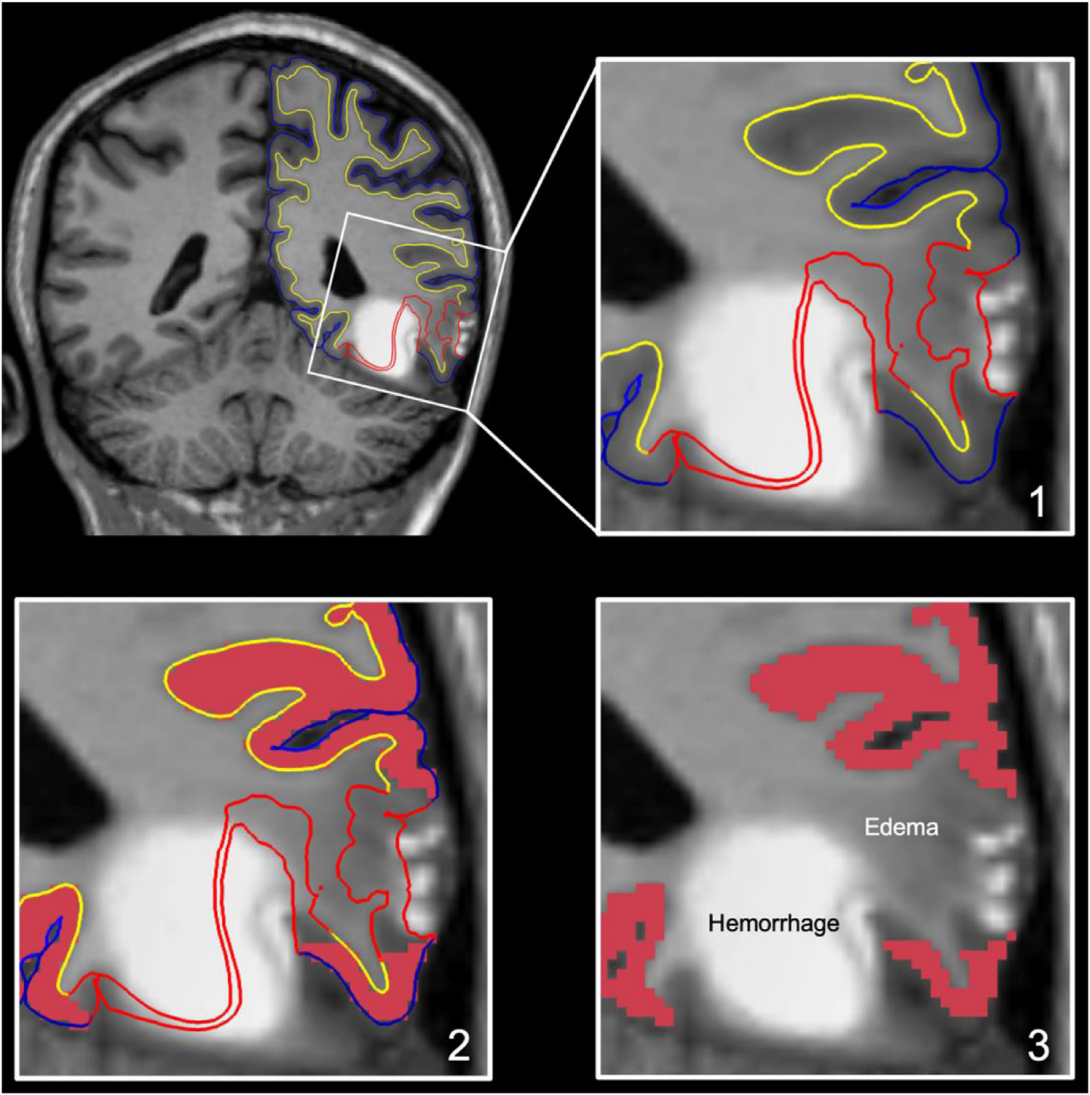
**Application of the Lesion Correction Tool to a Representative Patient with a Left Temporal Contusion from Acute Severe TBI.** Top left: FreeSurfer reconstructions of the cortical surface (blue line) and gray-white surface (yellow line) are used to visually identify regions where a cortical lesion caused surface inaccuracies (red lines). The image is shown in the coronal plane. Top right (step 1): a zoomed view of the square region shown in the top left image reveals the heterogeneous signal characteristics of the lesion, with the hemorrhagic component being hyperintense and the edematous component being hypointense. Bottom left (step 2): The apparently unlesioned cortex, represented by the tissue between the blue line (pial surface) and yellow line (gray-white junction), is filled in red. Bottom right (step 3): To correct for the inaccuracy in the surface label at the site of the lesion, we remove the volume of cortex within the lesion label and perform cortical volumetric measures that exclude the lesioned tissue.

**Fig. 7:**
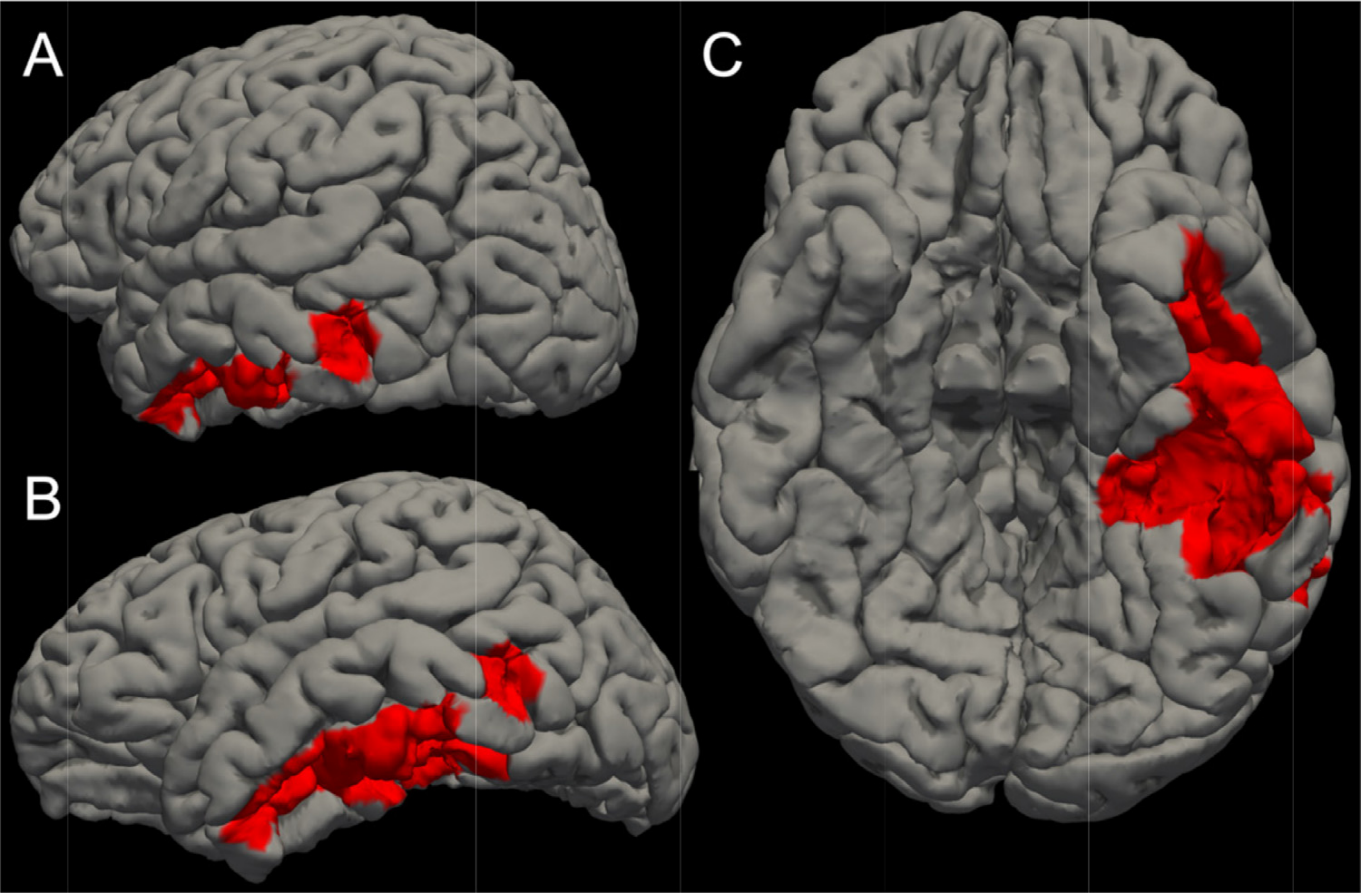
**Left Temporal Lesion on Cortical Surface of Representative Patient with Acute Traumatic Brain Injury.** We manually outlined the left temporal lesion shown in Fig. 6 by labeling inaccurate vertices on the cortical surface. This lesion label is shown in red from a left posterior oblique perspective (A), a left lateral perspective (B), and an inferior perspective (C). This red-labeled region of cortex would be removed from cortical volumetric analyses. The method can thus be readily applied to acute lesions, but as shown in Fig. 6, it is more challenging to accurately delineate the anatomic borders of acute lesions than it is for chronic lesions.

To demonstrate the potential challenges associated with applying this tool to patients with acute lesions, we applied the lesion correction tool to a representative patient with an acute left temporal contusion (Fig. 6, Fig. 7). As with chronic cortical lesions, FreeSurfer generates pial and gray-white surfaces at the site of the acute cortical lesion that do not reflect the underlying anatomy. However, unlike the clear delineation of lesioned from unlesioned cortex in the chronic patients studied here, visual delineation of lesioned cortex is challenging to perform in the representative acute patient due to mixed signal characteristics of the hemorrhagic (hyperintense) and edematous (hypointense) signal characteristics of the acute contusion (Fig. 6).

In summary, we demonstrate the impact of a new, semi-automated FreeSurfer-based lesion correction tool on cortical volumetric measures in 7 atlas-based functional networks, and we distribute this lesion correction tool to the academic community. We show that cortical lesions are not evenly distributed across networks, but rather preferentially affect the frontotemporal nodes of the limbic network. This lesion correction method can facilitate inclusive, unbiased investigation into the anatomic basis of neurological deficits in patients with TBI and other neuropsychiatric diseases associated with focal lesions.

## Declaration of Competing interest

The authors declare that they have no known competing financial interests or personal relationships that could have appeared to influence the work reported in this paper.

None of the authors has a conflicting financial interest. Dr. Fischl has financial interest in CorticoMetrics, a company whose medical pursuits focus on brain imaging and measurement technologies. His-interests were reviewed and are managed by Massachusetts General Hospital and Partners HealthCare in accordance with their conflict of interest policies.

## Appendix Supplementary materials

**Video 1. Overview of Network-based Lesion Overlap Analysis.** A left temporal lesion is shown in yellow for a representative patient. The yellow lesion mask represents all cortical vertices that were affected by the cortical lesion, as evidenced by inaccuracies in the FreeSurfer-generated cortical surface. Next, the 7-Network Yeo atlas is shown on the cortical surface, with the same network color-labeling shown in Figure 4. Finally, the portions of the Yeo networks that overlap the lesion mask are shown. This video demonstrates how a single lesion can overlap with multiple functional networks. Image, video 1

**Video 2. A Three-Dimensional View of Lesion Topology on the Cortical Surface**. The lesion topology map shown in the left panel of Figure 4 is displayed here in three dimensions. The anatomic regions most commonly affected by cortical lesions were the frontal and temporal lobes, particularly the frontal poles, temporal poles and orbitofrontal regions. Image, video 2

**Video 3. Visualization of Individual Lesion Topology on the Cortical Surface**. Motivated by prior efforts to map the cortical topology of traumatic contusions by Courville in 1942 \elsamp #x005B;22\elsamp #x005D;, we show the sequential overlapping of every cortical lesion identified in the 46 patients with lesions in this study. The video is designed in the style of Courville\elsamp #x0027;s classic lesion topology figure. Image, video 3
